# Fluoxetine treatment during the postpartal period may have short-term impacts on murine maternal skeletal physiology

**DOI:** 10.3389/fphar.2023.1244580

**Published:** 2023-11-23

**Authors:** Hannah P. Fricke, Chandler J. Krajco, Molly J. Perry, Lauren J. Brettingen, Lella A. Wake, Julia F. Charles, Laura L. Hernandez

**Affiliations:** ^1^ Endocrinology and Reproductive Physiology Program, University of Wisconsin-Madison, Madison, WI, United States; ^2^ Department of Animal and Dairy Sciences, University of Wisconsin-Madison, Madison, WI, United States; ^3^ Departments of Orthopedics and Medicine, Brigham and Women’s Hospital, Boston, MA, United States

**Keywords:** serotonin, SSRI, lactation, bone, calcium

## Abstract

Postpartum depression affects many individuals after parturition, and selective serotonin reuptake inhibitors (SSRIs) are often used as the first-line treatment; however, both SSRIs and lactation are independently associated with bone loss due to the role of serotonin in bone remodeling. Previously, we have established that administration of the SSRI fluoxetine during the peripartal period results in alterations in long-term skeletal characteristics. In the present study, we treated mice with either a low or high dose of fluoxetine during lactation to determine the consequences of the perturbation of serotonin signaling during this time period on the dam skeleton. We found that lactational fluoxetine exposure affected both cortical and trabecular parameters, altered gene expression and circulating markers of bone turnover, and affected mammary gland characteristics, and that these effects were more pronounced in the dams that were exposed to the low dose of fluoxetine in comparison to the high dose. Fluoxetine treatment during the postpartum period in rodents had short term effects on bone that were largely resolved 3 months post-weaning. Despite the overall lack of long-term insult to bone, the alterations in serotonin-driven lactational bone remodeling raises the question of whether fluoxetine is a safe option for the treatment of postpartum depression.

## 1 Introduction

Postpartum depression (PPD) is defined in the fifth edition of the Diagnostic and Statistical Manual of Mental Disorders (DSM-5) by the American Psychiatric Association as a major depressive episode that occurs either during pregnancy or the first 4 weeks after parturition (American Psychiatric Association, 2013). Symptoms of PPD can include a depressed mood, anhedonia, sleep disturbances, and suicidal ideation (American Psychiatric Association, 2013). Approximately 10%–15% of individuals are affected by PPD, and there are serious consequences associated with PPD for both parties of the parent–offspring dyad (Kroska and Stowe, 2020). There is evidence that PPD interferes with bonding between parent and child and is associated with increased negative interactions between parent and child when compared to non-depressed counterparts (Lovejoy, 1991). Some of the strongest risk factors for PPD include antenatal depression, antenatal anxiety, and a history of depression (Milgrom et al., 2008). The postpartum period often comes with a transitory change in mood that is commonly referred to as either the “baby blues” or the “postpartum blues,” and this transient change in mood can also increase an individual’s risk of developing PPD (Stowe and Nemeroff, 1995).

In the United States, selective serotonin reuptake inhibitors (SSRIs) are the most commonly prescribed class of antidepressants among peripartal individuals for the treatment of depression, as well as other serotonin-related psychiatric disorders (Nutt et al., 1999; Cooper et al., 2007; Davanzo et al., 2011). All SSRIs function by targeting the serotonin transporter, SERT, to prevent the reuptake of serotonin into the presynaptic nerve ending and thus increasing the amount of serotonin in the synaptic cleft (Raap and Van de Kar, 1999). Though they are designed to target SERT centrally, peripheral SERT is genetically identical to SERT found in the brain, and therefore, SSRIs also have an effect on peripheral serotonin (Coleman, Green, and Gouaux, 2016).

Serotonin, a biogenic monoamine synthesized from the essential amino acid tryptophan, has many roles centrally and peripherally. Centrally, serotonin is responsible for temperature regulation, sexual behaviors, mood, appetite, and sleep (Lucki, 1998). Peripherally, serotonin is involved in gastrointestinal motility, vasoconstriction, inflammation, bone homeostasis, and lactation (Rapport, Green, and Page, 1948; Matsuda et al., 2004; Bertrand, 2006; Yadav et al., 2008; Margolis et al., 2014). Tryptophan is first converted to 5-hydroxytryptophan (5-HTP) by tryptophan hydroxylase (TPH). Two different isoforms of TPH exist; TPH1 is expressed peripherally, while TPH2 is expressed centrally (Grahame-Smith, 1964; Walther et al., 2003). Next, 5-HTP is decarboxylated to form serotonin. From there, serotonin is primarily metabolized into an inactive form by monoamine oxidase (MAO). After parturition, tryptophan content is briefly increased in plasma, but there is a decrease in central tryptophan that is associated with a decrease in tryptophan transport across the blood-brain barrier (Baïlara et al., 2006). Along with a decrease in central tryptophan, there is also an increase in MAO-A binding early in the postpartum period (Sacher et al., 2010). Together, this provides evidence of a possible dysregulation of the serotonergic system shortly after parturition.

During lactation, the mammary gland orchestrates bone resorption in a serotonin-dependent manner in order to provide sufficient calcium to the offspring; over 6 months of exclusive breastfeeding, the maternal skeleton will lose 6%–10% of bone mass (Kovacs and Kronenberg, 1997). Over these 6 months, an average of 260 mg/L of calcium is transferred to the neonate via milk, and the maternal body will utilize calcium stored in bone to accommodate (Atkinson et al., 1995; Kovacs, 2016). As a litter-bearing species, over a 21-day lactation, rodents will lose approximately 20%–30% of their bone mass (Rasmussen, 1977; Kovacs and Kronenberg, 1997; Zeni, Di Gregorio, and Mautalen, 1999). Independently of lactation, SSRI usage has been associated with a decrease in bone mass and an increased fracture risk, and this may be due to the role of serotonin in bone remodeling (Richards et al., 2007; Tsapakis et al., 2012). In a rodent model, SERT inhibition in female mice resulted in a phenotype of decreased BMD and altered bone microarchitecture, which was demonstrated regardless of estrogen deficiency (Warden et al., 2008).

The skeleton is composed of two primary types of bone. Cortical bone composes approximately 80% of the adult skeleton and is largely responsible for stability (Ott, 2018; Bhatta and Jones, 2021). The rest of the skeleton is composed of trabecular bone, which is a more metabolically active type of bone that experiences a greater rate of turnover than cortical bone (Ott, 2018; Bhatta and Jones, 2021). Activation, resorption, reversal, and formation are the four phases of bone remodeling (Parfitt, 1988). The resorption phase is relatively short compared to the formation phase; the process of bone breakdown takes approximately 2–4 weeks, while bone building takes 4–6 months (Bhatta and Jones, 2021). The three primary cell types that are involved in bone remodeling are osteoclasts, osteoblasts, and osteocytes. Osteoblasts, bone-building cells, are derived from multipotent mesenchymal stem cells and osteocytes, bone breakdown cells, originate from the macrophage-monocyte cell lineage (Walker, 1973). Osteocytes, which are long-living mature osteoblasts, reside in the lacuna-canalicular system located within the mineralized matrix of bone (Ashique et al., 2017).

As the primary mechanoreceptors of bone, osteocytes communicate with osteoblasts and osteoclasts to orchestrate bone remodeling (Mohamed, 2008). Osteocytes can also participate in a physiological process known as osteocytic osteolysis, in which they act like osteoclasts and resorb minerals from the surrounding bone (Teti and Zallone, 2009; Wysolmerski, 2013). There are a few mechanisms in which lactation-related bone loss is thought to occur. Studies in animal models have shown increased osteoclast activity, which is associated with an insult to trabecular bone and a decrease in trabecular number (Kovacs, 2016). In humans, lactation has been associated with increased circulating CTX, which further establishes the link between increased osteoclast activity and bone loss during lactation (Kovacs, 2016). A second postulated mechanism for lactation-related bone loss is osteocytic osteolysis. Osteocytes are critical in promoting osteoclast activity via RANKL production, and it has been previously shown that signaling via PTHR1, the receptor shared by PTH and PTHrP, promotes osteocyte-specific RANKL production (O’Brien et al., 2008; Nakashima et al., 2011; Xiong et al., 2011; Xiong and O’Brien, 2012). Due to the relationship between lactation, SSRI usage, and bone loss, the aim of this current study was to examine the effect of different doses of fluoxetine during lactation and the implications on the maternal skeleton at the end of lactation and in the long term.

## 2 Materials and methods

### 2.1 Animals

Experiments were all performed under protocol number A005789-R01-A03 and were approved by the Research Animal Care and Use Committee at the University of Wisconsin–Madison. Female C57BL/6 mice were ordered from Jackson Laboratories at 5 weeks of age ± 3 days (stock #000664, Jackson Laboratories, Bar Harbor, ME). The mice were then housed in groups until they were housed separately beginning on the first day of pregnancy. The animals were housed in the Biochemistry vivarium, an environmentally controlled facility for biological research, at the University of Wisconsin–Madison. Mice were maintained at a temperature of 25°C and a humidity of 50%–60% on a 12-h light/dark cycle with food (Envigo-Teklad #2018) and water access *ad libitum*.

**FIGURE 1:**
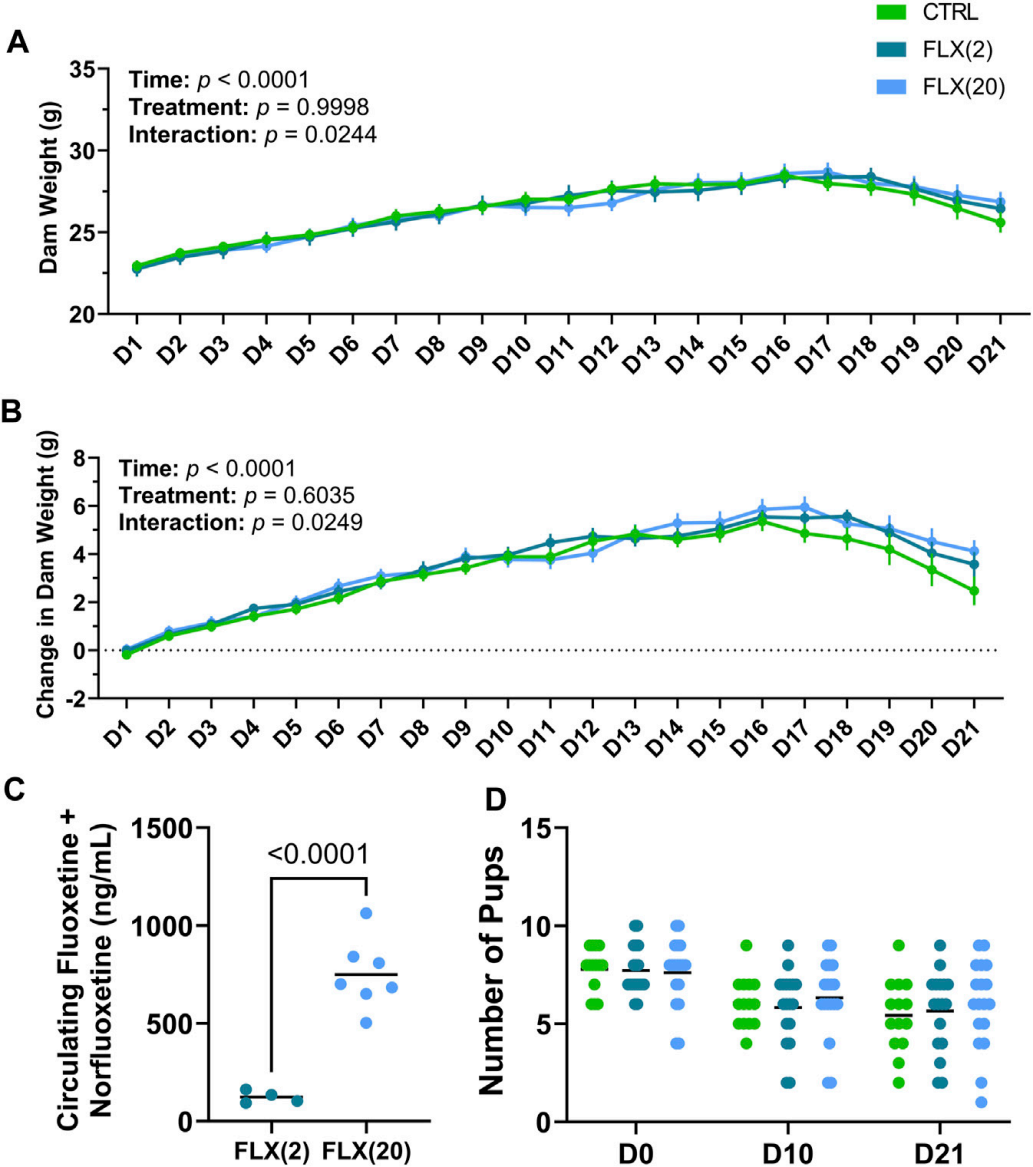
Fluoxetine administration during lactation had no overall treatment effect on dam weight throughout lactation, alter circulating FLX + NFLX concentrations based on the dose administered, and did not impact litter size at any point throughout lactation. C57BL/6J dams were administered sterile saline (*n* = 17), 2 mg/kg fluoxetine (*n* = 18), or 20 mg/kg fluoxetine (*n* = 19) daily. **(A)** Dam weight was measured daily at the time of treatment. **(B)** Dam weight was examined relative to the weight at parturition (D0). **(C)** The circulating concentrations of FLX + NFLX were measured in the dams at weaning (*n* = 4 2 mg/kg FLX; *n* = 7 20 mg/kg FLX). **(D)** The number of pups per litter was counted at parturition (D0), peak lactation (D10), and at weaning (D21). *p* < 0.05 is considered significant.

Starting at 6 weeks of age, the female mice were mated overnight with a C57BL/6 male obtained from Jackson Laboratories. The first day of pregnancy (E0) was determined by the presence of a vaginal mucus plug the next morning. The mice were monitored throughout gestation, and at parturition (D0), they were randomly assigned to receive sterile saline (*n* = 17), 2 mg/kg (*n* = 18), or 20 mg/kg (*n* = 19) of body weight of fluoxetine hydrochloride (#S6319; Sigma-Aldrich, St. Louis, MO, United States) reconstituted in sterile saline via intraperitoneal injection daily between the hours of 0800 and 0900. The treatment was administered from D0 through the end of lactation (D21). The dam weight number of pups and total litter weight were recorded daily at the time of treatment administration from D0 to D21. The litters were not standardized due to the inability to control for pup loss between treatment groups, but there was no significant difference in litter size at any point (Figure 1D). The dams were then either harvested at D21 (saline: *n* = 8; 2 mg/kg fluoxetine: n = 10; 20 mg/kg fluoxetine: *n* = 9) or aged out to 3 months post-weaning before being harvested (saline: *n* = 9; 2 mg/kg fluoxetine: *n* = 8; 20 mg/kg fluoxetine: *n* = 10). Previously, we demonstrated a link between a low dose of fluoxetine (2 mg/kg) during lactation and detrimental effects on the maternal skeleton. Because of the variability in dosage in the human population, we therefore further investigated the relationship between lactational fluoxetine administration and maternal bone by adding a cohort of dams dosed with the high dose of fluoxetine (20 mg/kg) and analyzing the data in a dose-dependent context. The data from both the control and low-dose fluoxetine cohorts are separately analyzed in an additional manuscript focused on the effects of low-dose fluoxetine during pregnancy, pregnancy and lactation, and lactation, while this study is focused on the effects of a low and high-dose of fluoxetine only during lactation (Fricke et al., 2023).

### 2.2 Sample collection

After a 6–7 h fasting period, blood samples were collected between the hours of 1400 and 1500. The blood sample was collected from the maxillary vein into Eppendorf tubes on E0, the beginning of lactation (D2), peak lactation (D10), D21, and 3 months post-weaning (3 MO). Blood was centrifuged at 1,500 g at 4°C for 20 min to isolate serum and serum was stored at −80°C until the time of assay.

At either D21 or 3 MO, dams were euthanized via carbon dioxide inhalation, and a cervical dislocation was performed afterward as a secondary form of euthanasia. The left femur was collected and fixed in 70% ethanol until Micro-computed tomography (micro-CT) analysis. The right femur, as well as the lower right mammary gland, were harvested and snap-frozen in liquid nitrogen and were stored at −80°C until the time of assay. The lower left mammary gland was collected and fixed overnight in a histological cassette at 4°C in 4% paraformaldehyde and then placed in 70% ethanol until embedded in paraffin.

### 2.3 DEXA analysis

Dual-energy x-ray absorptiometry (DEXA) was used to measure bone densitometry of the dams via a PIXImus2 Mouse Densitometer with the Lunar Piximus Software (GE Medical Systems, Madison, WI) as previously described (Lee et al., 2014). Before each session, quality control measurements were performed with a phantom. During each session, the mice were anesthetized via isoflurane inhalation. At 6 weeks of age, DEXA was performed as a baseline measurement and then was subsequently performed on D2, D10, D21, and 3 MO. Bone mineral density (BMD) of the femur and total body was measured, and analysis of the scans was performed with the Lunar Piximus Software using auto-thresholding.

### 2.4 Micro-CT analysis

Micro-CT was used to analyze the femurs of the dams. A Sanco Medical μCT 35 system with an isotropic voxel size of 7 μm was used, and scans were conducted in 70% ethanol. An x-ray tube potential of 55 kVp, an x-ray intensity of 0.145 mA, and an integration time of 400 ms were used. The femoral length of each femur was measured via digital calipers. The cancellous bone was measured via a selected region beginning 0.14 mm proximal to the growth plate and extending 1.4 mm proximally. Cortical and trabecular bone were distinguished using a semi-automated contouring approach. A selected region centered at the midpoint of the femur and 0.6 mm in length was used to measure cortical parameters. A global threshold that set the bone/marrow cutoff at 512 mgHA/cm^3^ for trabecular bone and 871.8 mgHA/cm^3^ for cortical bone was used to select the region of interest. Three-dimensional microstructural properties of the bone, which include parameters such as the bone volume fraction (BV/TV), midshaft bone volume fraction (M.BV/TV), trabecular thickness (Tb.Th), cortical thickness (C.Th), trabecular number (Tb.N.), and trabecular separation (Tb.Sp.) were calculated with software provided by the manufacturer. All calculations were reported according to consensus guidelines on rodent micro-CT (Bouxsein et al., 2010).

### 2.5 Assays

Serum calcium concentrations were measured via Cayman Chemicals Calcium Assay Kit (catalog no. 701220; Cayman Chemicals, Ann Arbor, MI) per manufacturer’s instructions. Samples were diluted 1:2 to fit within the standard curve. Serum serotonin concentrations were measured using the Beckman Coulter Enzyme Immunoassay Kit (catalog no. IM1749; Beckman Coulter, Vršovice, Czech Republic) per the manufacturer’s instructions. Serum samples were diluted 1:200 to fit within the standard curve. Serum collagen type 1 cross-linked C-telopeptide (CTX) concentrations were measured via RatLaps™ (CTX-I) Immunodiagnostics Systems enzyme immunoassay (catalog no. AC-06F1; Immunodiagnostics Systems, Tyne and Wear, United Kingdom) per manufacturer’s instructions. Serum procollagen I intact N-terminal (P1NP) concentrations were measured via Immunodiagnostics Systems enzyme immunoassay (catalog no. AC-33F1; Immunodiagnostics Systems, Tyne and Wear, United Kingdom) per manufacturer’s instructions. Samples were diluted 1:10 to fit within the standard curve. All assays had an intra-assay CV of <15% and inter-assay CV of <15%.

### 2.6 Mammary gland and femur RNA, RT-qPCR, and histology

TRI-Reagent (catalog no. NC9330796; Molecular Research, Cincinnati, OH) was used to extract RNA from the mammary gland and the femur, and RNA was reverse transcribed (1 μg) to cDNA via the Applied Biosystems High Capacity cDNA Reverse Transcription Kit (catalog no. 4368814; Applied Biosystems, Foster City, CA). The CFX96 Touch Real-Time PCR Detection System (Bio-Rad Laboratories, Rodeo, CA) was used to perform quantitative RT-PCR, and the reaction mixtures and cycling conditions were performed as previously described [43]. Primers were designed to span exon-exon junctions, and an optimal annealing temperature of 60°C was used. Amplification efficiencies were accepted within 95%–105%, and the primer specificity was determined by the presence of a single temperature dissociation peak. Primer sequences are listed in Table 1. The housekeeping parameter for the mammary gland was the geometric mean of *Rps15*, *Rps9*, *K8*, and *K14*, and the geometric mean of *Rps15* and *Hprt1* for the femur. Analysis was conducted using the 2^−ΔΔCT^ method (J. Laporta et al., 2013). Mammary glands were sectioned and stained with hematoxylin and eosin (H&E). All images were captured at 20x using QuPath-0.3.2 software on a Zeiss Axio Vert. 1 microscope.

**TABLE 1:** Primer sequences used for RT-qPCR.

Gene	Forward primer 5′ → 3′	Reverse primer 3′ → 5′
*Casp3*	CCA​AAT​GAG​AAA​GCT​GTC​AGG	TTG​AGG​TAG​CTG​CAC​TGT​GG
*Ccnd1*	TGA​TTC​TGG​CAC​ATT​CTT​GC	TCA​CCT​CTT​CCC​TCA​CAT​CC
*Gli1*	GGC​AGG​GAA​GAG​AGC​AGA​CT	ACTGCCTGCTGGGGAGTG
*Hprt1*	CTG​GTG​AAA​AGG​ACC​TCT​CG	AAC​TTG​CGC​TCA​TCT​TAG​GC
*Krt8*	ATC​GAG​ATC​ACC​ACC​TAC​CG	AAG​CCA​GGG​CTA​GTG​AGT​CC
*Krt14*	TCT​TGG​CGG​TGG​TAT​TGG​TGA​T	CAG​GCT​CTG​CTC​CGT​CTC​AAA​CT
*M-csf*	CGA​ATG​TTC​TCC​CAC​TTC​CT	TGG​ACA​ATC​AAA​GGC​TGA​GG
*Mcp1*	CCA​AAG​AAG​CTG​TAG​TTT​TTG	GGT​TCC​GAT​CCA​GGT​TTT​TA
*Mmp13*	CCG​AAC​TTA​ACT​TAC​AGG​ATT​G	GGT​GTC​ACT​CAG​ACC​AGA​CC
*Opg*	AAGCTGGAACCCCAGAGC	GTG​CTG​CAC​TTC​GTG​TGT​TT
*Orai1*	ACC​CCA​CGA​GCG​CAT​GCA​TC	GCT​TGG​TGG​GGC​TTG​GCT​GT
*Pmca2*	ACG​TAT​GGG​GAC​ACT​GAA​GC	TTG​CCC​AAA​AAT​CTG​TTT​CC
*Pthlh*	TTC​CTG​CTC​AGC​TAC​TCC​GT	GAT​GGA​CTT​GCC​CTT​GTC​AT
*Rank*	CAG​GAC​AGG​GCT​GAT​GAG​AG	CCG​CTA​GAG​ATG​AAC​GTG​GA
*Rankl*	GGA​GGA​TGA​AAC​AAG​CCT​TTG	ACA​TCC​AAC​CAT​GAG​CCT​TC
*Rsp9*	GGA​GAC​CCT​TCG​AGA​AGT​CG	GGG​GAT​CCT​TCT​CGT​CTA​GC
*Rps 15*	TTG​AGA​AAG​GCC​AAA​AAG​GA	GTT​GAA​GGT​CTT​GCC​GTT​GT
*Shh*	CTC​CGA​TGT​GTT​CCG​TTA​CC	GCC​TGG​CTC​TTT​CTC​TTC​CT
*Tnfα*	AGA​CCC​TCA​CAC​TCA​GAT​CAT	TCAGCCACTCCAGCTGCT
*Tph1*	TTC​ACC​ATG​ATT​GAA​GAC​AAC	TCC​GAC​TTC​ATT​CTC​CAA​GG
*Trap*	CGA​CAA​GAG​GTT​CCA​GGA​GA	TGC​CAA​GGT​GAT​CAT​GGT​TT

Abbreviations: *Casp3*, caspase-3; *Ccnd1*, cyclin D1; *Gli1*, GLI, family zinc finger 1; *Hprt1*, hypoxanthine phosphoribosyltransferase 1; *Krt8*, keratin 8; *Krt14*, keratin 14; *M-csf*, colony stimulating factor 1; *Mcp1*, monocyte chemoattractant protein-1; *Mmp13*, matrix metallopeptidase 13; *Opg*, osteoprotegrin; *Orai1*, calcium release-activated calcium modulator 1; *Pmca2*, plasma membrane Ca^2+^ ATPase, 1; *Pthlh*, parathyroid hormone like hormone; *Rank*, receptor activator of nuclear factor κΒ; *Rankl*, receptor activator of nuclear factor κΒ ligand; *Rps 9*, 40S ribosomal protein S9; *Rsp15*, 40S ribosomal protein S15; *Shh*, sonic hedgehog; *Tnfα*, tumor necrosis factor alpha; *Tph1*, tryptophan hydroxylase 1; *Trap*, tartrate-resistant acid phosphatase.

### 2.7 Statistics

All statistics were conducted with GraphPad Prism 9 (Version 9.5.1). Analyses between the three different treatment groups with multiple time points were conducted using a two-way ANOVA with Tukey’s multiple comparison test to analyze the differences between treatment groups. Analyses without the effect of time were performed using a one-way ANOVA with Tukey’s multiple comparison test to analyze the differences between treatment groups. When data were not normally distributed, a Kruskal-Wallis test was performed for nonparametric data. Outliers were determined via the ROUT method in GraphPad Prism and removed. For analyses, differences among means were considered significant if *p* < 0.05. All values are reported as mean ± SD.

## 3 Results

### 3.1 Fluoxetine administration during lactation does not alter pup mortality or have a treatment-specific effect on dam weight

In order to monitor the effect of fluoxetine administration on dam weight, dams were weighed daily at the time of dosing throughout lactation. Overall, there was a significant difference over time in both the gross dam weight (*p* < 0.0001) (Figure 1A) and the change in dam weight relative to the day of parturition (*p* < 0.0001), with dams increasing their body weight as lactation progressed (Figure 1B). Treatment did not affect gross dam weight or the change in dam weight, but there was an overall treatment-by-time interaction for both measurements in the control animals towards the end of lactation (*p* < 0.05 and *p* < 0.05, respectively). Circulating concentrations of fluoxetine (FLX) and its active metabolite, norfluoxetine (NFLX), were measured in both fluoxetine-treated groups (Figure 1C). There were no detectable levels of fluoxetine in the control animals (data not shown). In the FLX(20) group, there was a significantly increased concentration of circulating FLX + NFLX than the FLX(2) group (*p* < 0.0001). Finally, the litter size at parturition, peak lactation, and weaning was examined (Figure 1D). There were no significant differences between the number of pups per litter between any of the treatment groups.

### 3.2 Fluoxetine administration during lactation impacts the dam skeleton at weaning in a dose-dependent manner

**FIGURE 2:**
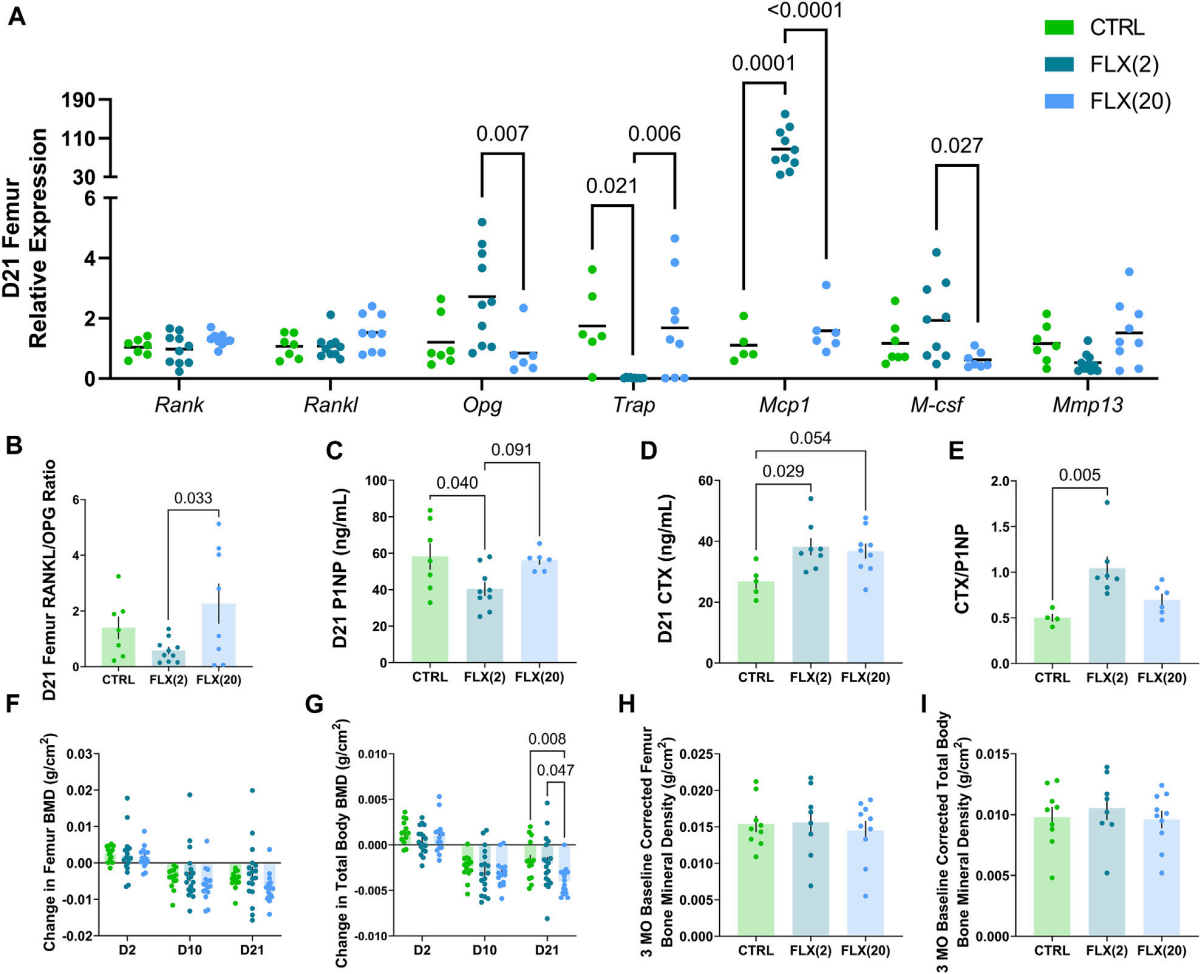
Either a low dose or a high dose of fluoxetine administered during lactation has differential effects on dam bone remodeling. C57BL/6J dams were administered either 2 or 20 mg/kg fluoxetine during lactation. At weaning, the femurs of each dam (*n* = 8 CTRL; *n* = 10 2 mg/kg FLX; *n* = 9 20 mg/kg FLX) were collected and RNA was extracted. **(A)** The relative gene expression in the femurs was evaluated. **(B)** The relative expression of RANKL to OPG was measured. **(C)** Circulating concentrations of procollagen 1 intact N-terminal propeptide (P1NP) were measured. **(D)** Circulating concentrations of carboxy-terminal collagen crosslinks (CTX) were measured. **(E)** The ratio of circulating CTX to P1NP was evaluated. **(F,G)** The change in BMD of the femur and the total body relative to the end of pregnancy were measured via DEXA (*n* = 17 CTRL; *n* = 18 2 mg/kg; *n* = 19 20 mg/kg). **(H,I)** At 3 months post-weaning, the change in BMD of the femur and the total body was evaluated relative to the end of lactation (*n* = 9 CTRL; *n* = 8 2 mg/kg; *n* = 10 20 mg/kg). *p* < 0.05 is considered significant.

The relative gene expression of dam femurs was measured at weaning to analyze the effect of either 2 mg/kg or 20 mg/kg fluoxetine administration on the dam bone (Figure 2A). In the femur, there was no significant difference in Rank or Rankl expression in any groups, but the expression of Opg was significantly increased in the FLX(2) group compared to the FLX(20) group (*p* < 0.01). The relative expression of Trap, a marker of bone resorption, was decreased in the FLX(2) group compared to both the control dams (*p* < 0.05) and the FLX(20) group (*p* < 0.01). Conversely, Mcp1, an important factor in osteoclastogenesis, was upregulated 85-fold in the FLX(2) group compared to the control dams (*p* < 0.001) and the FLX(20) dams (*p* < 0.0001). M-csf, a promoter of osteoclast proliferation and differentiation, was upregulated in the FLX(2) dams compared to the FLX(20) dams (*p* < 0.05). There were no significant differences in the expression of Mmp13, a mediator of bone remodeling. Further, the ratio of RANKL/OPG relative expression, an indicator of bone breakdown activity to bone building activity, was decreased in the FLX(2) group compared to the FLX(20) dams (*p* < 0.05) (Figure 2B).

To further explore the relationship between fluoxetine administration and skeletal outcomes in the dams, circulating markers of bone remodeling were measured in the serum at weaning. Procollagen 1 intact N-terminal propeptide (P1NP), a circulating marker of bone formation, was decreased in the FLX(2) group compared to the control dams (*p* < 0.05) (Figure 2C). Carboxy-terminal collagen crosslinks (CTX), a marker of bone resorption, was increased in the FLX(2) group compared to the controls (*p* < 0.05) and tended to be higher in the FLX(20) group compared to the controls (*p* < 0.1) (Figure 2D). The ratio of circulating P1NP to CTX was next examined (Figure 2E). Similar to the RANKL/OPG ratio, the CTX/P1NP ratio also measures the rate of bone breakdown to bone formation. There was a significant increase in the FLX(2) dams compared to the controls (*p* < 0.01). Finally, DEXA was used to examine the BMD of the femur and the total body throughout lactation (Figures 2F, G) and at 3 months post-weaning (Figures 2H, I). The change in BMD throughout lactation are shown relative to the end of pregnancy (E17.5), while the 3MO BMD measurements were relative to the BMD measured at the end of lactation. There were no significant differences in femur BMD at any point throughout lactation between any groups. In the total body measurements, however, there was a decreased change in BMD in the FLX(20) group compared to the controls (*p* < 0.01) and the FLX(2) group (*p* < 0.05). There were no significant differences in the change in 3 MO BMD of either the femur or the total body between any of the groups.

Micro-CT was used to measure cortical and trabecular skeletal parameters of the femur at either weaning or 3 months post-weaning (Table 2). In the cortical bone, there was an overall time effect in the cortical thickness (*p* < 0.0001), the periosteal perimeter (*p* < 0.0001), BMD (*p* < 0.0001), tissue mineral density (TMD) (*p* < 0.0001), and length (*p* < 0.0001). In the trabecular bone, there was an effect of time on the bone volume/trabecular volume (BV/TV) (*p* < 0.0001), trabecular number (*p* < 0.0001), trabecular spacing (*p* < 0.0001), BMD (*p* < 0.0001), TMD (*p* < 0.0001), and connectivity density (*p* < 0.0001). There was an effect of treatment on the cortical thickness (*p* < 0.01), cortical BMD (*p* < 0.01), and trabecular TMD (*p* < 0.01). There was no interaction of time and treatment in the trabecular bone, but there was an interaction in the cortical area (*p* < 0.05) and cortical BMD (*p* < 0.05).

**TABLE 2:** Cortical and trabecular skeletal parameters evaluated by micro-CT in animals exposed to either saline, 2 mg/kg fluoxetine, or 20 mg/kg fluoxetine at weaning (*n* = 8 CTRL; *n* = 10 2 mg/kg FLX; *n* = 9 20 mg/kg FLX), or at 3 months post-weaning (*n* = 9 CTRL; *n* = 8 2 mg/kg; *n* = 10 20 mg/kg).

		Measurements						*p*-value		
		D21			3 MO					
		CTRL	FLX (2 mg/kg)	FLX (20 mg/kg)	CTRL	FLX (2 mg/kg)	FLX (20 mg/kg)	Time	Treatment	Interaction
Cortical	Cortical Thickness (mm)	0.139 ± 0.007	0.134 ± 0.009	0.125 ± 0.012	0.184 ± 0.007	0.183 ± 0.009	0.177 ± 0.009	<0.0001	0.0024	0.4178
	Periosteal perimeter (mm)	8.910 ± 0.331	8.626 ± 0.259	9.130 ± 0.307	8.527 ± 0.166	8.509 ± 0.387	8.286 ± 0.242	<0.0001	0.2367	0.0018
	Ct.ar (mm^2^)	1.778 ± 0.073	1.700 ± 0.086	1.835 ± 0.078	1.803 ± 0.067	1.788 ± 0.113	1.741 ± 0.125	0.7972	0.2486	0.0157
	BMD (mg Hg/cm^3^)	444.6 ± 19.46	435.0 ± 26.02	391.2 ± 42.44	571.1 ± 14.66	575.7 ± 23.74	568.6 ± 18.50	<0.0001	0.0029	0.0135
	TMD (mg Hg/cm^3^)	1198.2 ± 13.55	1192.1 ± 10.52	1186.1 ± 21.25	1244.5 ± 13.53	1244.8 ± 7.07	1240.6 ± 11.74	<0.0001	0.2164	0.6510
	Length (mm)	15.716 ± 0.196	15.498 ± 0.406	15.759 ± 0.306	16.059 ± 0.380	16.169 ± 0.150	16.007 ± 0.393	<0.0001	0.8711	0.1583
Trabecular	BV/TV (%)	5.904 ± 1.389	5.275 ± 1.448	4.838 ± 1.126	2.369 ± 0.731	2.230 ± 1.031	2.144 ± 0.582	<0.0001	0.2185	0.5188
	Tb.N. (1/mm)	3.644 ± 0.198	3.585 ± 0.156	3.595 ± 0.304	2.432 ± 0.180	2.570 ± 0.296	2.507 ± 0.150	<0.0001	0.8687	0.4251
	Tb.Sp. (mm)	0.275 ± 0.016	0.279 ± 0.012	0.280 ± 0.025	0.412 ± 0.035	0.391 ± 0.046	0.398 ± 0.024	<0.0001	0.6932	0.3981
	Tb.Th. (mm)	0.035 ± 0.003	0.034 ± 0.002	0.034 ± 0.004	0.034 ± 0.012	0.033 ± 0.003	0.034 ± 0.006	0.7944	0.7797	0.9357
	BMD (mg Hg/cm^3^)	94.31 ± 15.31	88.15 ± 16.82	81.88 ± 12.69	55.80 ± 8.58	54.85 ± 13.48	53.45 ± 6.42	<0.0001	0.2282	0.4980
	TMD (mg Hg/cm^3^)	970.9 ± 14.28	952.2 ± 14.93	951.1 ± 21.01	1005.7 ± 14.02	988.1 ± 18.56	989.9 ± 22.10	<0.0001	0.0054	0.9415
	Conn. Density (1/mm^3^)	115.641 ± 26.92	117.203 ± 54.11	105.019 ± 40.57	23.056 ± 10.29	28.172 ± 17.03	28.004 ± 10.36	<0.0001	0.8412	0.7417

Abbreviations: Ct.ar, cortical area; BMD, bone mineral density; TMD, tissue mineral density; BV/TV, bone volume fraction; Tb.N., trabecular number; Tb.Sp., trabecular spacing; Tb.Th., trabecular thickness; Conn. density, connectivity density. Data are presented as mean ± SD and analyzed using two-way ANOVA for treatment and time. *p* < 0.05.

**FIGURE 3:**
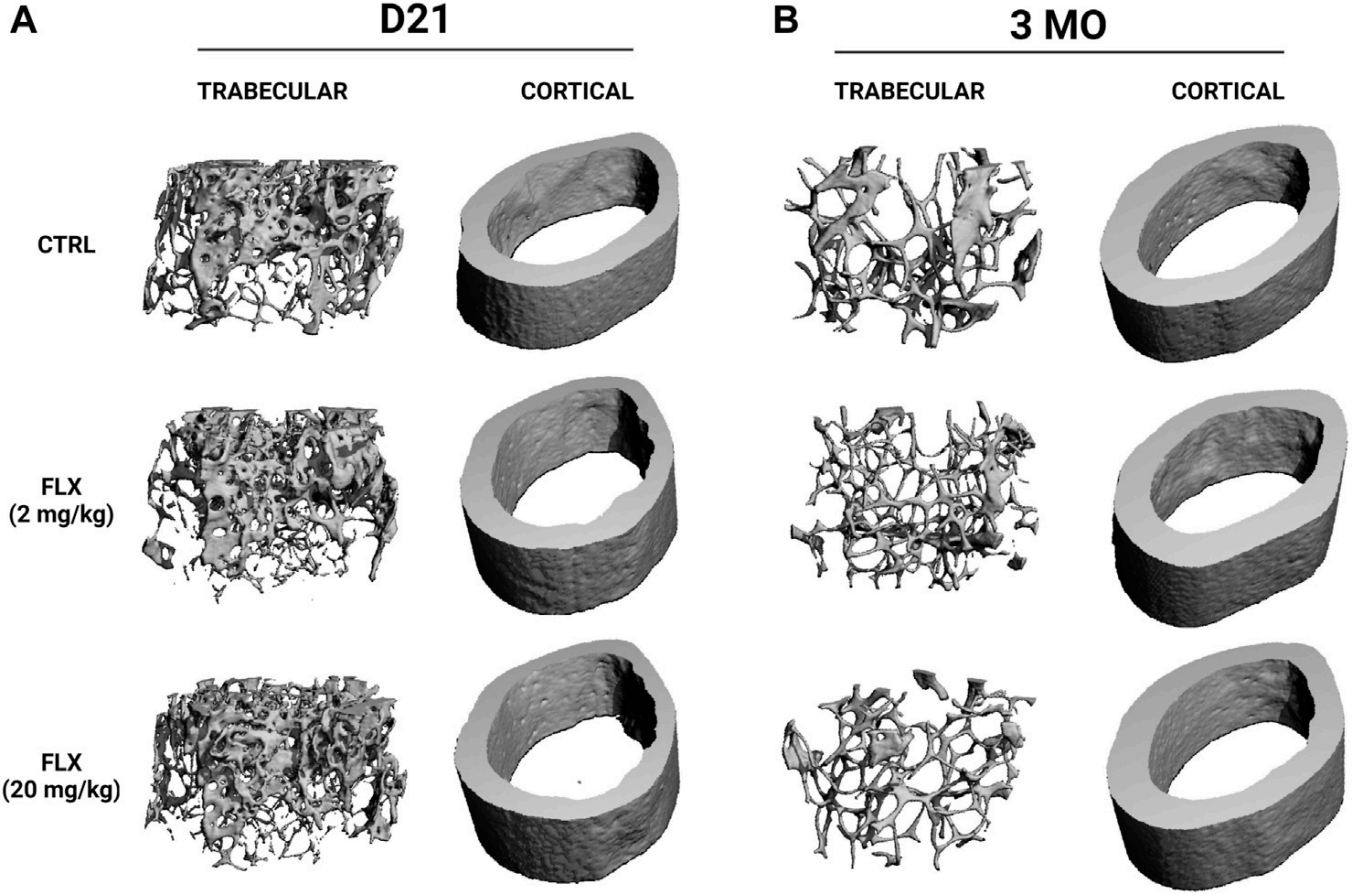
Representative images of the microarchitecture of trabecular and cortical bone of dams dosed with saline, 2 mg/kg fluoxetine, or 20 mg/kg fluoxetine during lactation only at weaning and 3 months post-weaning. **(A)** Three-dimensional images of distal femur trabecular and cortical bone at weaning (D21). **(B)** Three-dimensional images of distal femur trabecular and cortical bone at 3 months post-weaning (3 MO).

At D21, there was a significant decrease in cortical thickness in the FLX(20) group compared to the control group (*p* < 0.01). Further, the periosteal perimeter and cortical area were increased in the FLX(20) group compared to the FLX(2) group (*p* < 0.01 and *p* < 0.01, respectively). Cortical BMD was also decreased in the FLX(20) dams compared to both the control dams (*p* < 0.001) and the FLX(2) dams (*p* < 0.01), but no significant differences in any cortical parameters between groups at 3 MO. However, there were no significant differences between groups in any of the trabecular parameters at D21 or 3 MO. Representative images of the cortical and trabecular bone of all groups at both D32 and 3 MO in Figure 3.

### 3.3 Fluoxetine administration during lactation has a dose-dependent effect on gene expression of the mammary gland and circulating serotonin, but not calcium

**FIGURE 4:**
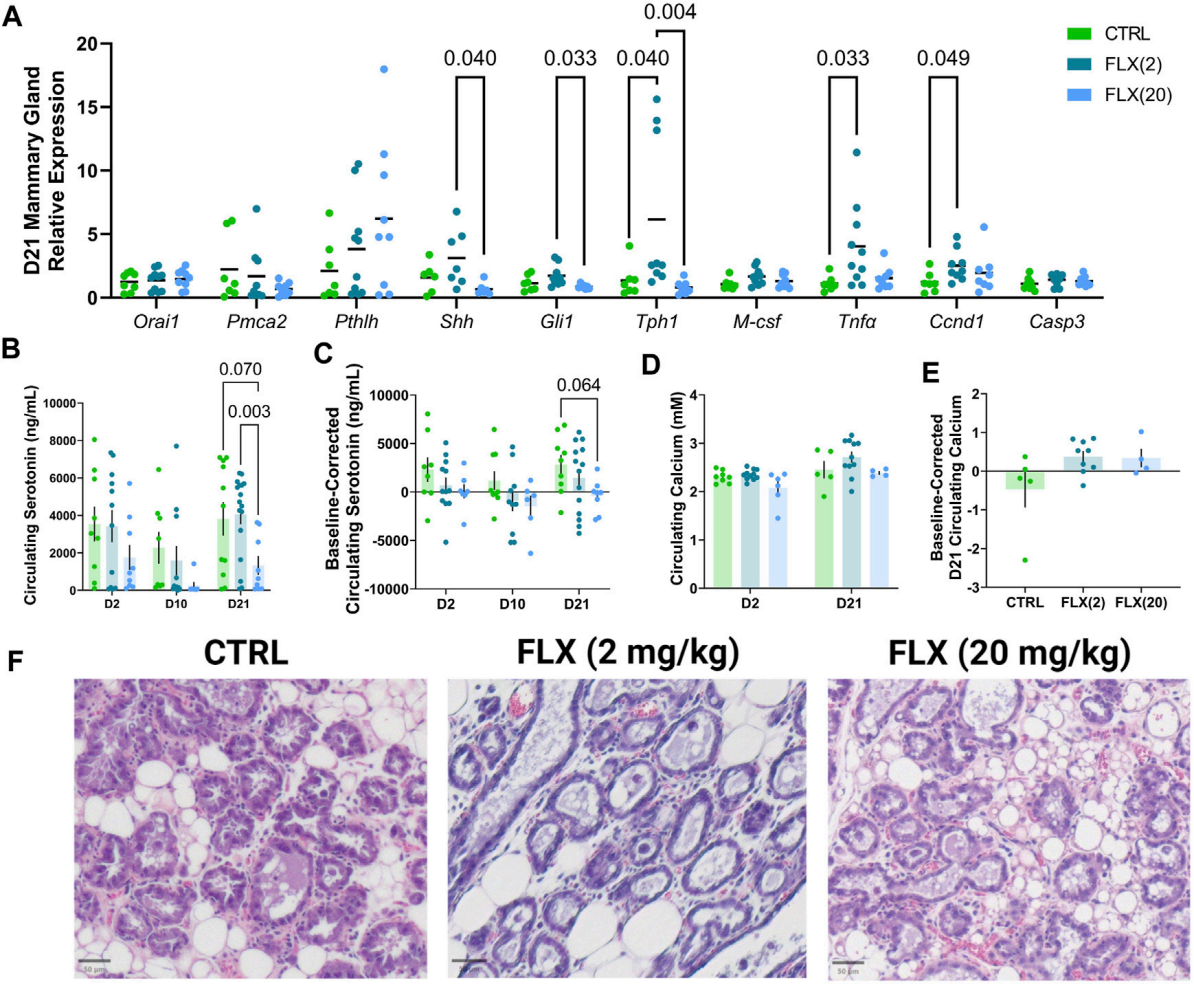
Fluoxetine administration during lactation had a dose-dependent impact on relative gene expression in the mammary gland, as well as in circulating serotonin concentrations at the end of lactation. C57BL/6J dams were administered saline (*n* = 8), 2 mg/kg FLX, (*n* = 10), or 20 mg/kg FLX (*n* = 9) daily during lactation. **(A)** At weaning, the mammary gland was collected to measure relative gene expression. **(B)** Serum was collected from the dams at the beginning, middle, and end of lactation. Circulating serotonin concentrations were then measured in all dams (*n* = 17 CTRL; *n* = 18 2 mg/kg; *n* = 19 20 mg/kg). **(C)** The change in serotonin concentrations relative to the serotonin concentrations at the end of pregnancy was measured. **(D)** Circulating calcium concentrations at the beginning and end of lactation were measured. **(E)** The circulating calcium at the end of lactation relative to the concentrations at the beginning of lactation was evaluated. **(F)** The right inguinal mammary gland was harvested from dams at weaning, fixed in 4% paraformaldehyde, and used for histological examination. Histological hematoxylin and eosin stained mammary glands at weaning.

Due to the role of the mammary gland in orchestrating bone remodeling during lactation, the relative mRNA expression in the mammary gland was measured at D21 (Figure 4A). There was no difference in the expression of Orai1, a calcium channel essential for calcium entry into mammary epithelial cells, or Pmca2, a calcium transporter, between groups. Further, there was no difference in the expression of Pthlh, the gene that encodes parathyroid hormone-related protein (PTHrP), which is an important regulator of calcium liberation from the bone by the mammary gland. There was a significant decrease in the expression of Shh and Gli1, components of the serotonin-driven signaling pathway that orchestrates bone remodeling by the mammary gland during lactation, in the mammary glands of the FLX(20) dams compared to the FLX(2) dams (*p* < 0.05 and *p* < 0.05, respectively). The expression of Tph1, the rate-limiting enzyme in serotonin synthesis, was upregulated in the FLX(2) dams compared to both the control dams (*p* < 0.05) and the FLX(20) dams (*p* < 0.01). The relative expression of Tnfα and Ccnd1, which are both involved in mammary epithelial proliferation, was upregulated in the FLX(2) group compared to the control dams (*p* < 0.05 and *p* < 0.05, respectively).

Next, circulating serotonin concentrations in the dam serum were measured at D21 (Figure 4B). There were no differences in circulating serotonin concentrations at D2 or D10, but it was significantly decreased in the FLX(20) group in comparison to the FLX(2) group (*p* < 0.01). Circulating serotonin was then analyzed as the change in serotonin concentration compared to the baseline, which was the end of pregnancy (Figure 4C). Similarly, there was a trend for the FLX(20) dams to have a decreased change in circulating serotonin compared to the control group (*p* < 0.1). Circulating calcium concentrations at the beginning and end of lactation were measured, as well as the circulating serotonin concentrations at the end of lactation relative to the levels at the beginning of lactation (Figures 4D, E). However, there were no observed differences between groups. Mammary gland structure was visualized via H&E (Figure 4F).

## 4 Discussion

Herein, we demonstrated that fluoxetine administration alters lactation-related bone remodeling and that there is a differential impact depending on whether a low or high dose was administered. Despite the established role of serotonin in eating habits and satiety, there were no differences in either gross dam weights or the change in dam weights relative to the beginning of lactation (Figures 1A, B) (Voigt and Fink, 2015). When looking at the level of the bone, there was an effect on the gene expression of markers of osteoclastogenesis and osteoclast activity that were exhibited in the dams that were administered the low dose of fluoxetine, but not the high dose. Further, there was a decreased rate of bone resorption in terms of the RANKL/OPG ratio, but not the CTX/P1NP ratio in the FLX(2) treatment group. Further, in the low dose, P1NP was decreased and CTX was increased; however, only the dams that were given the high dose of fluoxetine exhibited a decrease in the change in total body BMD at the end of lactation. Despite the changes in bone at weaning, there were no changes in the relative femoral or total body BMD 3 months post-weaning, which is in contrast to what we have observed previously in mice that were dosed with the 20 mg/kg dose of fluoxetine during the entire peripartal period (Weaver et al., 2018). At weaning, there appeared to be a greater impact of lactational fluoxetine on the cortical bone than trabecular bone, and this was primarily seen in the high-dose dams. In the 20 mg/kg dams, the cortical thickness and cortical BMD were decreased, while the periosteal perimeter and cortical area were increased. However, none of these differences between groups were seen at 3 months post-weaning.

In this experiment, the finding that cortical bone was affected to a greater degree than trabecular bone was not expected. Kaya and colleagues postulated that osteocytic osteolysis affects the composition of the local bone matrix, which then had the potential to affect the properties of cortical bone (Kaya et al., 2017). They demonstrated that lactation-driven osteocytic osteolysis marginally increased the osteocyte lacunar-canalicular space without altering the mineral content of the matrix (Kaya et al., 2017). Along with RANKL, osteocytes also produce M-CSF, which similarly promotes osteoclastogenesis (Zhao et al., 2002; Nakashima et al., 2011; Xiong et al., 2011). In the present study, *mcsf* mRNA expression in the femur was downregulated in the high dose of fluoxetine compared to the low dose, but neither differed significantly from the controls. Despite the close link between RANKL and M-CSF, there were no differences in RANKL expression. The relative expression of OPG, however, was upregulated in the low dose mice at the end of lactation compared to the high-dose (Figure 2A). This is significant, as it has been previously established that mice with a deficiency in OPG have a higher rate of bone turnover (Mizuno et al., 1998; Koide et al., 2013). This may, in part, explain the decrease in the RANKL/OPG ratio of gene expression in the femur and the decrease in circulating P1NP in the low-dose animals, given the inverse relationship between OPG and the rate of bone turnover.

Interestingly, the relative expression of TRAP, a phenotypic marker of osteoclast activity, was decreased in the low dose animals compared to both the controls and the high-dose animals. Conversely, however, the relative expression of *Mcp1*, an important mediator of osteoclastogenesis, was highly upregulated in the low dose animals. In the context of cancer, PTHrP has been shown to upregulate the expression of *mcp1* (Ricarte et al., 2018). In the mammary gland, there was no significant difference in the expression of *Pthlh*, the gene that encodes PTHrP, however, the circulating PTHrP in the dams was not measured, and the relative expression was examined at the end of lactation. Our lab has previously established that at peak lactation, there is an increase in *Pthlh* expression in the mammary gland when treatment is initiated prepartum and with the 20 mg/kg dose of fluoxetine, and thus, future research should incorporate different time points throughout lactation in order to fully determine the effect of fluoxetine at both a high and a low dose on mammary gland gene expression (Weaver et al., 2018). In rodents, circulating levels of PTHrP have been associated with an increase in circulating markers of bone resorption and a subsequent decrease in bone mass during lactation, and in humans, increased circulating PTHrP was correlated with bone loss (Sowers et al., 1996; VanHouten and Wysolmerski, 2003).

Two independent studies first characterized the role of serotonin in bone metabolism a little over two decades ago. Firstly, Bliziotes et al. (2001) found that SERT and various serotonin receptors were expressed in osteoblastic cells. In the same year, Westbroek et al. described the presence of the 5-HT_2B_ receptor in fetal bone tissue and murine osteoblast cultures (Westbroek et al., 2001). Battaglino et al. (2004) later demonstrated, in a cell culture model, that blocking SERT via fluoxetine resulted in osteoclast formation inhibition and a correlated decrease in the expression of osteoclast markers such as TRAP. Interestingly, the decrease in *trap* expression was consistent with our findings, but only at the low dose. Further, it has been previously demonstrated that serotonin signaling via the 5-HT_2B_ receptor plays an important role in osteoblast recruitment and proliferation, and fluoxetine acts on the 5-HT_2B_ receptor (Collet et al., 2008; Peng et al., 2014). In Wistar rats, treatment with an intermediate (8.2 mg/kg) dose of fluoxetine for 40 days resulted in a decrease in P1NP, but not CTX levels (Kumar et al., 2018). When dosed with fluoxetine during lactation, our study showed that mice exhibited a decrease in P1NP, but an increase in CTX at a low dose, and no significant difference in P1NP or CTX at a high dose. Our previous work also has shown that mice dosed with a high dose of fluoxetine throughout the entire peripartal period had a decrease in circulating P1NP, but no change in CTX, at weaning (Weaver et al., 2018). Of an important note, a previous study demonstrated that dosing mice with 20 mg/kg of fluoxetine for 3 weeks resulted in a decreased bone resorption, while treatment for 6 weeks resulted in a decrease in bone formation, suggesting a difference in short-term versus long-term dosing (Ortuño et al., 2016). This leads to the conclusion that there is either a dose-dependent effect, a lactational effect, or a combination of the two on markers of bone turnover in a rodent model, and further research should be focused on separating what occurs during these pivotal periods. Pregnancy and lactation, as well as dosing effects, appear to lead to differential effects on bone metabolism.

Along with changes in the bone, there were also changes observed in the mammary gland. At weaning, expression of TPH1 was greatly increased in the low-dose group compared to both the control group and the high-dose group, which suggests an increase in serotonin synthesis. Further, there was significantly more circulating serotonin at the end of lactation in the low-dose dams compared to the high-dose, which further alludes to an upregulation of serotonin synthesis in the low-dose treatment paradigm. Typically, SSRI results in reduced circulating serotonin concentrations due to the inhibition of SERT, which did not appear to occur in FLX(2), suggesting that the length of dosing, as well as the dose, did not impact peripheral serotonin concentrations. During lactation, in the mammary gland, PTHrP secretion is driven by serotonin via activation of the canonical hedgehog signaling pathway (Hernandez et al., 2012; Laporta et al., 2014). Serotonin induces this pathway by altering the methylation patterns of the sonic hedgehog promoter, which then activates PTHrP synthesis (Laporta et al., 2014). Here, in the high-dose dams, we observed downregulation of *shh* and *gli1* expression, a member of the canonical hedgehog signaling pathway, compared to the low-dose dams, but not the control dams. These data suggest that lower doses of fluoxetine treatment may not impact hedgehog and PTHRP as observed with a 20 mg/kg dose (Laporta et al., 2013; Weaver et al., 2018). The alterations in circulating serotonin, demonstrated by both serum serotonin and variation in *Tph1* expression in the mammary gland, appear to be differentiallyimpacted by the lower dose compared to the high dose, likely due to the fact the length and dose of fluoxetine administered did not inhibit SERT to the extent that the 20 mg/kg dose did. This could be due to, in part, a partial inhibition of SERT at the low dose of fluoxetine, and a more complete inhibition at the higher dose. Additionally, there was not a significant decrease in the amount of circulating serotonin at the beginning or mid lactation in either dose, but that is not entirely unsurprising given the short length of dosing at the earlier time points. The expression of *Tnfα*, an important factor in mammary gland involution, and *Ccnd1*, which is required for progression of the cell cycle, are both upregulated in the low-dose, but not the high-dose group (Levy et al., 2010; Xuan et al., 2022). This suggests that the low dose of fluoxetine, but not the high, impacts mammary gland involution. This is consistent with our previous findings in which sertraline, another member of the SSRIs, was shown to hasten mammary gland involution at the end of lactation (Sheftel et al., 2022).

There are several limitations to this study that warrant discussion. Firstly, there is a substantial difference in the among of lactation-associated bone loss between rodents and humans. Rodents, as a litter-bearing species, lose approximately 20–30 percent of their bone mass over a 21-day lactation (Kovacs and Kronenberg, 1997; Qing et al., 2012; Woodrow et al., 2006). Humans, as a monotocous species, lose between 5–10 percent over 3–6 months of lactation (Kalkwarf and Specker, 1995; Polatti et al., 1999; Kovacs, 2011; Bremeck et al., 2015; Bjørnerem et al., 2017). When comparing serum fluoxetine levels at different doses, Dulawa et al. (2004) established pharmacologically relevant doses in rodents compared to the serum levels of fluoxetine in humans. In this experiment, the doses we chose, while still pharmacologically relevant, represent a very low and a very high dose compared to human dosing. Future studies should include intermediate doses, such as 10 mg/kg, to further explore the spectrum of dose effects on fluoxetine administration during lactation. This is important because fluoxetine is unique in that it exhibits a nonlinear kinetic profile, and so there is a disproportionate increase in circulating fluoxetine concentrations with higher doses. This may be due, in part, to the self-inhibitory action of fluoxetine; fluoxetine is primarily demethylated to norfluoxetine in the liver by CYP2D6 and CYP2C9, and it is inhibitory to CYP2D6, which results in the uniquely nonlinear kinetic profile (Hiemke and Härtter, 2000; von Moltke et al., 1997). Thus, intermediate doses are potentially critical in understanding the mechanism of fluoxetine modulation of bone remodeling during lactation. Along with different dosages, female reproductive hormones should be evaluated in future experiments. There is an established relationship between serotonin and hormones such as estrogen, progesterone, prolactin, and oxytocin, and therefore, the potential relationship between serotonin modulation and critical hormones during the peripartal period should also be investigated (Kamberi et al., 1971; Kordon et al., 1973; Bethea, 1993; Uvnäs-Moberg et al., 1999; Lu et al., 2004; Mottolese et al., 2014). Finally, the effect of lactational fluoxetine was primarily examined at the end of weaning and 3 months post-weaning. In order to fully elucidate the relationship between fluoxetine, lactation, and bone, the phenotypic effects of fluoxetine administration should be analyzed throughout lactation.

In conclusion, lactational fluoxetine exposure has a transient dose-specific effect on the dams at both the level of the mammary gland and the bone. There were no differences seen in the change in femoral or total body BMD, or any cortical or trabecular parameters between groups at 3 months post-weaning, which differs from our previously reported results of a high dose of fluoxetine administered during both gestation and lactation (Weaver et al., 2018). Whether this is due to the window of exposure spanning the entire peripartal period versus solely lactation or if it is more relevant to an acute versus chronic dosing paradigm is unclear. There are also dramatic differences in the effects of a high versus a low dose of fluoxetine. This likely is due to the inability of the low dose to inhibit SERT fully. However, we did not utilize any intermediate doses in the present study, so we are unable to determine if this is due to the nonlinear kinetic profile of fluoxetine or a possible biphasic dose-response curve is unclear. Further work incorporating different doses of fluoxetine or different members of the SSRI class of antidepressants should be conducted in order to more fully elucidate the effect of lactational antidepressant usage on the maternal skeleton and the possible deleterious effects of pharmacological treatment of PPD.

## Data Availability

The original contributions presented in the study are included in the article/Supplementary Material, further inquiries can be directed to the corresponding author.
